# Effects of lumacaftor—ivacaftor therapy on cystic fibrosis transmembrane conductance regulator function in F508del homozygous patients with cystic fibrosis aged 2–11 years

**DOI:** 10.3389/fphar.2023.1188051

**Published:** 2023-05-30

**Authors:** Julian Berges, Simon Y. Graeber, Susanne Hämmerling, Yin Yu, Arne Krümpelmann, Mirjam Stahl, Stephanie Hirtz, Heike Scheuermann, Marcus A. Mall, Olaf Sommerburg

**Affiliations:** ^1^ Division of Pediatric Pulmonology and Allergology and Cystic Fibrosis Center, Department of Pediatrics, University of Heidelberg, Heidelberg, Germany; ^2^ Department of Translational Pulmonology, Translational Lung Research Center Heidelberg (TLRC), German Center for Lung Research (DZL), University of Heidelberg, Heidelberg, Germany; ^3^ Department of Pediatric Respiratory Medicine, Immunology and Critical Care Medicine and Cystic Fibrosis Center, Charité—Universitätsmedizin Berlin, Corporate Member of Freie Universität Berlin and Humboldt-Universität zu Berlin, Berlin, Germany; ^4^ Berlin Institute of Health at Charité—Universitätsmedizin Berlin, Berlin, Germany; ^5^ German Center for Lung Research (DZL), Associated Partner Site, Berlin, Germany

**Keywords:** CFTR modulator therapy, cystic fibrosis (CF), CFTR function, lumacaftor/ivacaftor, intestinal current measurement (ICM), sweat chloride

## Abstract

**Rationale:** Lumacaftor/ivacaftor was approved for the treatment of patients with cystic fibrosis who are homozygous for F508del aged 2 years and older following positive results from phase three trials. However, the improvement in CFTR function associated with lumacaftor/ivacaftor has only been studied in patients over 12 years of age, while the rescue potential in younger children is unknown.

**Methods:** In a prospective study, we aimed to evaluate the effect of lumacaftor/ivacaftor on the CFTR biomarkers sweat chloride concentration and intestinal current measurement as well as clinical outcome parameters in F508del homozygous CF patients 2–11 years before and 8–16 weeks after treatment initiation.

**Results:** A total of 13 children with CF homozygous for F508del aged 2–11 years were enrolled and 12 patients were analyzed. Lumacaftor/ivacaftor treatment reduced sweat chloride concentration by 26.8 mmol/L (*p* = 0.0006) and showed a mean improvement in CFTR activity, as assessed by intestinal current measurement in the rectal epithelium, of 30.5% compared to normal (*p* = 0.0015), exceeding previous findings of 17.7% of normal in CF patients homozygous for F508del aged 12 years and older.

**Conclusion:** Lumacaftor/ivacaftor partially restores F508del CFTR function in children with CF who are homozygous for F508del, aged 2–11 years, to a level of CFTR activity seen in patients with CFTR variants with residual function. These results are consistent with the partial short-term improvement in clinical parameters.

## Introduction

Cystic fibrosis (CF) is an autosomal recessive disorder caused by pathogenic variants in the cystic fibrosis transmembrane conductance regulator (CFTR) gene. The defect affects the CFTR protein, an ion channel in the apical membrane of epithelial cells, and leads to a multisystem disease that primarily affects the lungs, pancreas and gastrointestinal tract (Elborn, 2016). F508del is the most common CFTR variant and is present in more than 60% of all CF patients worldwide on at least one allele causing a defect in the folding and trafficking of the CFTR protein, resulting in impaired epithelial chloride and bicarbonate transport (Mall and Hartl, 2014). The lung disease remains the major cause of morbidity and mortality in CF and is caused by impaired mucociliary clearance leading to mucus plugging, infection with opportunistic germs, inflammation and subsequently the development of bronchiectasis (Bell et al., 2020; Mall et al., 2020).

The CFTR corrector lumacaftor and the potentiator ivacaftor were proven in combination (LUM/IVA) as first causative treatment designed to rescue CFTR protein function in patients with CF homozygous for the F508del variant (F/F). LUM/IVA was approved in the European Union (EU) on 18 November 2015 for the treatment of patients with CF being F/F over the age of 12 years. For pediatric use, the EU marketing authorization was extended on 08 January 2018 for children aged 6–11 years and on 15 November 2018 for children aged 2 years and older. It is therefore currently the only available CFTR modulator for children with CF being F/F between 2 and 5 years.

Approval for patients over 12 years of age was based on the results of two large phase-3 trials (Wainwright et al., 2015). However, in these studies, LUM/IVA resulted in only a modest improvement in FEV1%predicted, ranging from 2.6 to 4.0 percentage points compared to placebo, with considerable heterogeneity between study participants. The subsequent 96-week open-label extension study showed that the main benefit of LUM/IVA lay in the reduction of respiratory exacerbations, suggesting a lower annual loss of lung function (Konstan et al., 2017). Authorization for the pediatric patients with CF being F/F was based on a decrease of the Lung Clearance Index (LCI) in patients aged 6 years and older of 1.09 compared to placebo after 24 weeks and a decrease in sweat chloride concentrations (SCC) of 20.8 mmol/L (Ratjen et al., 2017). In patients aged 2–5 years, a drop in SCC of −31.7 mmol/L from baseline after 24 weeks was shown, alongside a significant increase in body weight and body-mass-index (BMI) (McNamara et al., 2019). The latter phase-3 trial lacked a placebo treated control group, thus clinical findings appear less convincing, especially as the placebo-controlled study in 6–11-year-olds had precisely not been able to demonstrate a significant increase in BMI (Ratjen et al., 2017). However, the considerable decrease in SCC hinted at a substantial role of CFTR biomarker testing for the prediction of long-term benefits of LUM/IVA. Since the therapeutic concept of CFTR modulator therapy aims at mitigating the long-term loss of pulmonary function, direct evaluation of CFTR function under treatment became desirable. In addition to SCC measurement, nasal potential difference (nPD) and intestinal current measurement (ICM) are considered functional tests that can be used as even more sensitive diagnostic tools for determining CFTR activity. With these tests, our group was able to demonstrate for 53 patients with CF being F/F above the age of 12 years that LUM/IVA rescued CFTR function in nPD to a level of 10.2% and in ICM to 17.7% of normal, respectively (Graeber et al., 2018).

The first aim of this study was therefore to investigate the efficacy of LUM/IVA correction of CFTR function in sweat ducts and intestine in pediatric patients aged 2–11 years. In order to do this, we designed a sub-study as part of an ongoing prospective longitudinal observational study at our CF center and measured the CFTR biomarkers sweat chloride concentration and ICM as well as clinical outcomes like BMI, spirometry and LCI at baseline and 8–16 weeks after starting treatment with LUM/IVA. As a second aim, we wanted to determine the relationship between partial rescue of F508del-CFTR function and potential short-term clinical response to LUM/IVA.

## Methods

Routine clinical data were collected using standard methods and are not described further. Additional information on the special studies briefly described below can be found in the online Supplementary Material.

### Study design and participants

The prospective mono-center sub-study was conducted within a prospective longitudinal observational study in patients with CF at the CF center of the University Children’s Hospital Heidelberg, Germany, belonging to the German Centre for Lung Research (DZL). Both the sub-study and the above-mentioned overarching prospective longitudinal observational study were approved by the ethics committee of the Medical Faculty of the University of Heidelberg (S-489/2015 and S-370/2011, respectively). Patients who met the inclusion criteria were eligible to participate in the sub-study. This was the case if they were at least 2 but less than 11 years old, homozygous for the CFTR variant F508del (F/F), had no previous exposure to LUM/IVA and were willing to adhere to a stable medication regimen for the duration of the study according to the regulatory approved labelling and prescribing information for the appropriate age and weight. Exclusion criteria included acute respiratory infection or pulmonary exacerbation at baseline, or concomitant disease that may pose an additional risk to LUM/IVA administration or confound the results of the study. Participation in another clinical intervention trial also led to exclusion from our study. Written informed consent was obtained from all parents or legal guardians of participating patients. In all patients participating in the sub-study, sweat chloride concentrations and ICM (Graeber et al., 2015) as well as clinical parameters including anthropometry, clinical blood chemistry, LCI, lung function test parameters (Quanjer et al., 2012) were determined at baseline and 8–16 weeks after starting the therapy with the individually approved LUM/IVA dose. The time window for the follow-up measurements was chosen to facilitate the planning of study visits under real-life conditions and had also been chosen in our previous study with this objective when examining patients over 12 years of age (Graeber et al., 2018).

### Evaluation of CFTR activity

The assessment of CFTR function in this sub-study was done exclusively by measurement of SCC and ICM using rectal biopsies.

#### Sweat chloride concentration (SCC)

The SCC measurement was performed according to the German guideline “Diagnose der Mukoviszidose” (Naehrlich et al., 2013) and the guidelines of the Clinical and Laboratory Standards Institute (LeGrys, 2019). Samples were collected with the Macroduct^®^ system (Model 3700, Wescor, Logan UT, United States). Sweat chloride concentration was measured in a minimum volume of 30 μL using a chloridometer (KWM 20 Chloridometer, Kreienbaum, Langenfeld, Germany).

#### Intestinal current measurement (ICM)

The ICM was performed as described before (Graeber et al., 2015) at baseline and after initiation of LUM/IVA. Endoscopic forceps biopsies were performed on rectal mucosa. Tracings were evaluated by two independent readers (J.B. and S.H.) and a mean of collected scores was used as a final outcome. Quality criteria were response to test stimulation with carbachol, stability of recordings as well as baseline and final tissue resistance. Normal CFTR activity was defined as response to cAMP dependent stimulation obtained in age-matched non-CF control subjects [age: 8.7 ± 5.1 (mean ± SD)], and percentage of normal CFTR function was calculated for each patient with CF by dividing the individual cAMP-induced I_eq_ of the patient by the median cAMP induced I_eq_ of the age-matched control group as previously described (Graeber et al., 2018).

### Lung clearance index (LCI)

The LCI was determined using the Exhalyzer D system (EcoMedics) with the multiple breath washout (MBW) test. 100% oxygen was used to wash residual nitrogen from the lungs using a mouthpiece as an interface (Rowe et al., 2014). All measurements were analysed using Spiroware 3.3.1 (EcoMedics) (Wyler et al., 1985; Stahl et al., 2014; Graeber et al., 2021). The upper limit of normal was set at 7.1 (Wyler et al., 1985).

### Statistical data analysis

The sample size calculation was based on a previous study by our group in adult F508del/F508del CF patients (Graeber et al., 2018). We calculated the sample size for the change in IBMX/forskolin in a paired *t*-test assuming a nominal type I error of 0.05 and a power of 0.8, resulting in an estimated sample size of 10 patients. Data were analyzed with the aid of statistical software Prism version 6 (Graph Pad Software Inc., San Diego, CA, United States) and Grapher version 8 (Golden Software LLC, Golden, CO, United States). Data were presented as median with interquartile range (IQR) or mean with standard deviation (SD). Parametric data were evaluated according to paired *t*-tests. Non-parametric data were evaluated by Wilcoxon matched pairs signed rank test. A *p*-value below 0.05 was considered significant.

## Results

### Characteristics of study population

**FIGURE 1:**
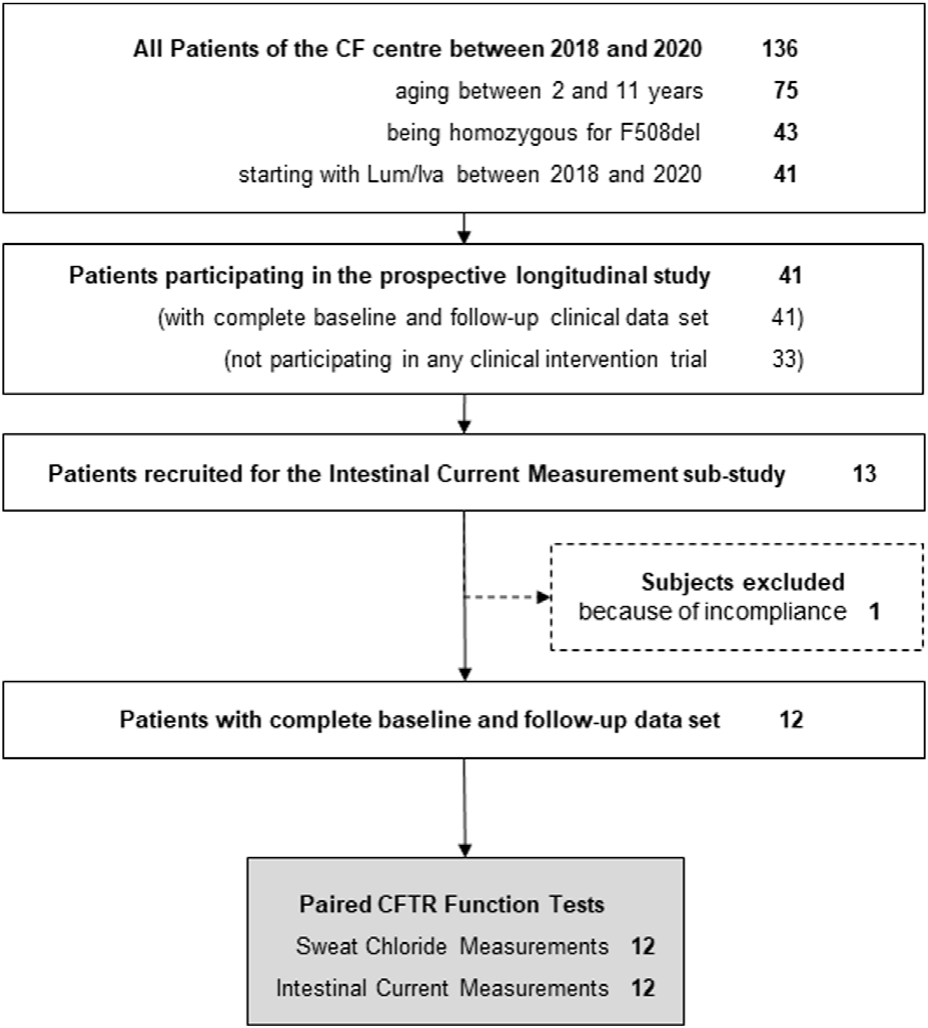
Flow chart of study design.

Between 2018 and 2020, 41 patients with CF who were F/F aged between 2 and 11 years, and who started with LUM/IVA, were treated at our CF centre. All these patients were part of an overarching prospective longitudinal study that provided the clinical data set for this sub-study. 8 of these 41 patients participated in other clinical intervention trials during this time, leaving 33 patients eligible for this sub-study (Figure 1). Of these, only 13 were finally recruited into the present sub-study, as a number of the patient’s parents declined additional invasive testing as part of a trial on an approved drug. The 13 recruited patients received both the initial and follow-up ICM examination. One patient had to be excluded because he did not take LUM/IVA on the day of the second ICM, leaving 12 participants in the present ICM sub-study (Figure 1). From all 12 patients, 4 biopsies were measured for ICM at both baseline and follow-up (Figure 1). Anthropometric and clinical parameters before and during LUM/IVA treatment were also available for all 12 patients. Due to the young age of two patients, pulmonary function tests were only available for the remaining 10 patients. Table 1 shows in column “Baseline” the clinical characteristics at baseline of the sub-cohort of 2–11-year-old CF patients recruited for the ICM sub-study.

**TABLE 1:** Patient characteristics at baseline and course of selected clinical parameters under LUM/IVA therapy (*n* = 12, if not stated otherwise).

Clinical characteristic	Baseline	LUM/IVA therapy	Change between baseline and LUM/IVA	*p*-value
Number of patients	12			
Age (years)	7.8 ± 2.7 (2.0–11.7)	8.2 ± 2.5 (3.5–11.9)		
Sex, female, n (%)	5 (42%)			
BMI z-score	−0.19 ± 0.80 (−1.6 to 1.2)	0.05 ± 1.02 (−1.7–2.0)	0.24 ± 0.69 (−0.3)	0.2500
Weight percentile	44.0 ± 29.7 (4.0–92.0)	50.3 ± 32.2 (5.0–97.0)	6.33 ± 10.87	**0.0195**
LCI, *n* = 10	8.1 ± 1.4 (5.9–11.2)	7.3 ± 0.8 (6.1–8.8)	−0.83 ± 0.96	**0.0332**
FEV1% predicted, *n* = 10	95.53 ± 15.72 (69.30–119.70)	91.38 ± 13.76 (68.0–113.5)	−4.15 ± 5.34	**0.0363**
VCmax % predicted, *n* = 10	94.61 ± 13.15 (75.80–109.20)	92.54 ± 13.43 (76.20–115.0)	−2.07 ± 4.61	0.1897
FEV1/VCmax % predicted, *n* = 10	99.94 ± 7.63 (87.10–111.6)	97.85 ± 9.78 (84.60–109.0)	−2.09 ± 6.15	0.3106
MEF25% predicted, *n* = 10	92.0 ± 47.44 (33.40–172.6)	82.0 ± 43.26 (27.50–160.0)	−10.0 ± 36.74	0.4117

Definition of abbreviations: BMI, body mass index; FEV1, forced expiratory volume in 1 s; LCI, lung clearance index; VCmax, maximum vital capacity; MEF25, mean expiratory flow at 25% of capacity. Baseline refers to the time point of clinical evaluation before start with LUM/IVA, date of ICM, might deviate if diagnostic ICM, was performed in early childhood. Data are shown as mean ± SD (range).

Highlighted in bold are p-values below 0.05.

### LUM/IVA improves CFTR function in sweat ducts and intestinal epithelia in patients with CF being F/F aged 2–11 years

**FIGURE 2:**
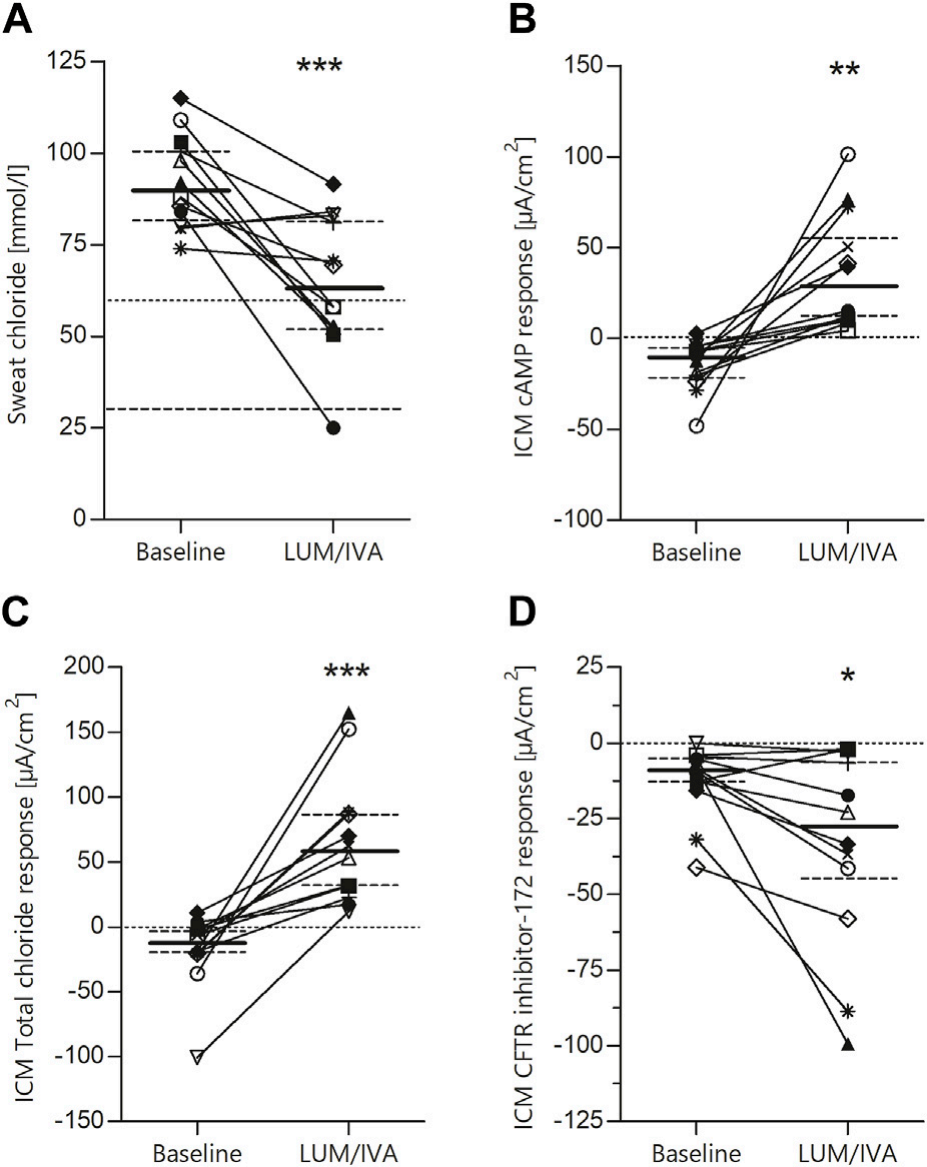
Significant improvement of CFTR function in F508del homozygous patients by LUM/IVA treatment. Paired values of individual study subjects before and after the start of LUM/IVA treatment for sweat chloride [**(A)**; median baseline 90.0, IQR 83.0–101.1; median LUM/IVA 63.8, IQR 52.0–82.0; median of difference—26.8 IQR 12.9–46.9; *p*-value 0.0006], cAMP response in rectal biopsies measured in the Ussing chamber [**(B)**; median baseline −10.7, IQR −22.9 to −4.9; median LUM/IVA 27.3, IQR 10.1–67.0; median of difference 34.0, IQR 18.9–70.8; *p*-value 0.0015], total chloride response considering intestinal current after stimulation with carbachol [**(C)**; median baseline—12.6, IQR −20.2 to −1.9; median LUM/IVA 57.8, IQR 29.5–87.3; median of difference 63.7, IQR 40.7–109.4; *p*-value 0.0003] and change of intestinal current after treatment with the CFTR inhibitor 172 [**(D)**; median baseline—9.0, IQR −13.5 to −5.1; median LUM/IVA −28.2, IQR −45.6 to −5.6; median of difference −14.5, IQR −29.2 to −2.7; *p*-value 0.0237]. Statistical evaluation by means of paired t-tests. Solid lines represent the median and dashed lines represent the 25th and 75th percentiles.

**FIGURE 3:**
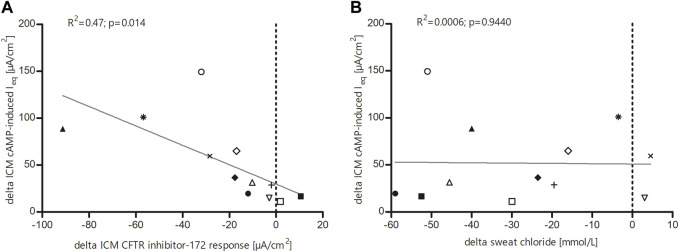
Linear regression analysis **(A)** between cAMP response and change in intestinal current after treatment with the CFTR inhibitor 172 and **(B)** between the different CFTR function surrogates sweat chloride and ICM cAMP response. **(A)** Significant correlation of individual study subjects’ values within the ICM measurements of cAMP response and change in intestinal current after treatment with the CFTR inhibitor-172. **(B)** Relationship between change in ICM cAMP response and change in sweat chloride levels in 12 F508del homozygous children under LUM/IVA treatment. Change in ICM response did not correlate with change in sweat chloride levels.

To determine the effects of LUM/IVA on CFTR function *in vivo*, we measured SCC and ICM at baseline and after initiation of the therapy. SCC at baseline were 90.0 mmol/L (IQR 83.0–101.1), thus in the typical CF range. After start of LUM/IVA treatment a decrease by 26.8 mmol/L (IQR 12.9 to 46.9; *p*-value 0.0006) was observed (Figure 2A). Looking at the subjects individually, two patients had no improvement in SCC and one patient had only a very moderate improvement in SCC. Assignment of distinct symbols for each patient in this study’s graphs allows for visual traceability throughout the different CFTR function tests (*cf.* below). ICM measurements showed a significant improvement in CFTR function for the investigated 2–11-year-old patients. At baseline, the absence of relevant Cl-secretion was confirmed upon cAMP-mediated stimulation (Figure 2B). In these samples, small negative currents (I_eq_ = −10.7 µA/cm^2^, IQR −22.9 to −4.9) could be observed after cAMP-induced stimulation, which is consistent with previous results and most likely due to concomitant K^+^ secretion (Veeze et al., 1991; Graeber et al., 2018). After initiation of LUM/IVA, cAMP-mediated stimulation increased intestinal currents to 27.3 µA/cm^2^ (IQR 10.1 to 67.0; *p*-value 0.0015), corresponding to a rescue of CFTR function in the rectal mucosa to a level of 30.5% of normal (IQR 9.9–46.5). Examination of the total chloride response, which comprises cAMP-mediated stimulation augmented by cholinergic co-activation, confirmed these findings with a median of differences of 63.7 µA/cm^2^ (IQR 40.7 to 109.4; *p*-value 0.0003; Figure 2C). Total chloride response under LUM/IVA corresponded to 16.0% of normal total chloride response in age matched controls (IQR 8.2–24.2). It is interesting to note that the ICM improved under LUM/IVA even in those patients who did not show any improvement in the SCC at the same time. To confirm that improved ion secretion indeed depended on CFTR function, selective inhibition with the CFTR inhibitor-172 subsequent to cAMP-mediated and cholinergic stimulation was performed. The resulting ΔI_eq_ at baseline and under therapy were compared. It could be shown that the inhibitory potential under LUM/IVA was significantly bigger than before (−28.2 µA/cm^2^ vs. −9.0 µA/cm^2^; *p*-value 0.024; Figure 2D), proving that cAMP-induced I_eq_ responses reflected CFTR-dependent chloride secretion. This was confirmed by a positive correlation (R2 = 0.47, *p*-value 0.014) between cAMP response and the degree of consecutive CFTR inhibition by the CFTR inhibitor-172 (Figure 3A).

### Substantial concordance but no quantitative correlation among CFTR biomarkers

Next, we investigated the relationship between sweat chloride secretion and cAMP-induced chloride secretion in the ICM, because the improvements seen there after starting LUM/IVA therapy raised the question of a possible correlation between these CFTR biomarkers, such that one may predict the other. However, no correlations were observed between the change in SCC and the change in ICM response (R2 = 0.0006; *p* = 0.9440). Nonetheless, a high concordance was observed for the two CFTR biomarkers in 10 out of 12 patients (Figure 3B). Furthermore, all patients with CF being F/F in our sub-study showed improvements in at least one of the CFTR biomarkers.

### Short-term measures of clinical outcomes show a slight benefit from LUM/IVA treatment

We investigated the response of selected clinical parameters to improved CFTR activity following LUM/IVA therapy. Conventional lung function tests showed no relevant functional improvement in a simple before and after comparison (Table 1). In the sub-cohort of 12 patients we studied, there was even a significant decrease in FEV1%predicted by −4.15 percentage points (Table 1). However, in contrast to conventional pulmonary function test, a significant improvement in LCI was observed which decreased by −0.7 with LUM/IVA treatment (median differences, *p*-value 0.0371; Table 1, Supplementary Figure S1A). Linear regression analysis revealed no quantitative correlation between the two CFTR biomarkers SCC and ICM cAMP response and LCI (sweat chloride: R2 = 0.004; *p* = 0.86, ICM: R2 = 0.06; *p* = 0.50). However, there was moderate concordance in 6 out of 10 patients for sweat chloride concentration and good concordance in 8 out of 10 patients for ICM cAMP response (Supplementary Figures S1B, C).

Regarding weight gain in patients treated with LUM/IVA, there was an increasing trend in BMI z-score, but this was not significant (*p*-value 0.25). However, it was notable that the tendency to cross the age-dependent weight percentiles upwards was significant in our cohort (median difference 4; median pre-treatment 40.5, IQR 18.3–61.8; median post-treatment 48.0, IQR 25.8–79.5; +6.3 percentiles; *p*-value 0.02; Table 1).

## Discussion

This is the first study to investigate the effects of combination therapy with the CFTR modulators lumacaftor and ivacaftor (LUM/IVA) on CFTR function in organ systems such as sweat glands and the intestine using the CFTR biomarkers SCC and ICM in a cohort of 12 children with CF with two F508del less than 12 years of age and to correlate these results with short-term clinical outcome.

LUM/IVA is currently the only available CFTR modulator for children with CF who are F/F between 2 and 5 years of age and, along with tezacaftor-ivacaftor (TEZ/IVA) and the recently approved elexacaftor-tezacaftor-ivacaftor (ELX/TEZ/IVA), the only CFTR modulator therapy for children with CF who are F/F older than 6 years of age [e.g., (Ramsey and Welsh, 2017; De Boeck, 2020)]. Phase-3 studies have investigated the safety and clinical efficacy of LUM/IVA therapy in both adults and children (Wainwright et al., 2015; Konstan et al., 2017; Ratjen et al., 2017; McNamara et al., 2019) and in a previous prospective multicentre study (Graeber et al., 2018), our group was able to evaluate the effect of LUM/IVA on CFTR function in different organ systems in patients with CF who are F/F over 12 years of age using the CFTR biomarkers SCC, nPD and ICM. The present prospective monocenter observational study evaluates the *in vivo* effects of LUM/IVA on CFTR function in children aged 2–11 years by measurement of SCC and ICM, as nPD cannot yet be performed safely without sedation and with sufficient quality in infants and preschool children. Furthermore, unlike nPD, ICM can achieve standardized conditions even in very young children.

In our study in a cohort of 12 patients, the assessment of functional correction by the CFTR biomarkers SCC and ICM showed for the first time that LUM/IVA therapy results in consistent partial rescue of CFTR function in different organs in children with CF who are F/F between the ages of 2 and 11 years (Figures 2A–D). For the CFTR biomarker SCC our results show an average reduction of −26.8 mmol/L, which is comparable to the previously mentioned phase-3 studies where the reduction was −32 mmol/L in children aged 2–5 years (McNamara et al., 2019) and −20 mmol/L in children aged 6–11 years (Milla et al., 2017; Ratjen et al., 2017). Compared with our previous multicenter study in patients over 12 years of age, in which we measured a reduction in SCC of −18 mmol/L (Graeber et al., 2018), and an earlier phase-2 study in adult patients, in which the decrease in SCC with LUM/IVA therapy was only −8 to −10 mmol/L (Boyle et al., 2014), this suggests that the efficacy of LUM/IVA in improving CFTR function in the sweat glands may be more pronounced in children than in adults. In this respect, the comparison with the other approved CFTR modulators TEZ/IVA and ELX/TEZ/IVA is also interesting. Recently, we were able to report results on the improvement of CFTR activity with these therapies in F/F CF patients older than 12 years. In 18 patients treated with a TEZ/IVA therapy for the first time a reduction in SCC of −13.0 mmol/L could be shown (Graeber et al., 2022a). After these patients were switched to ELX/TEZ/IVA, SCC decreased further by −50.5 mmol/L (Graeber et al., 2022a). Compared to baseline, ELX/TEZ/IVA resulted in a mean reduction in SCC of −61.0 mmol/L (Graeber et al., 2022a).

The ICM measurements performed in our sub-study cohort of children with CF being F/F from 2 to 11 years showed a rescue of cAMP-mediated stimulation of CFTR activity in the rectal epithelium to a level of about 30% of normal (Veeze et al., 1991). This is higher than demonstrated in our previous study in patients with CF who are F/F over 12 years of age, where an improvement in CFTR function to 18% was shown (Veeze et al., 1991; Graeber et al., 2018). Notably, the rescue of CFTR activity in the present study in children was also above the level of functional improvement of about 25% maximum correction shown for LUM/IVA combination therapy in in vitro studies on primary bronchial epithelial cultures from F/F CF patients (Van Goor et al., 2011). In comparison, TEZ/IVA showed slightly worse results in the age group over 12 years, where an improvement in cAMP-mediated stimulation of CFTR activity of about 14% was seen in the 18 patients included in the aforementioned study (Graeber et al., 2022a). However, after switching these patients to ELX/TEZ/IVA, this value increased to 46% of normal CFTR activity (Graeber et al., 2022a). These results are comparable to patients with CF with a Gly551Asp mutation treated with ivacaftor monotherapy, resulting in improvements of 52% of normal CFTR activity in ICM studies (Graeber et al., 2015).

Our ICM studies show that LUM/IVA combination therapy improves CFTR activity in F/F CF patients aged 2–11 years to levels comparable to the lower range of levels in patients with CF and CFTR variants with residual CFTR function (Hirtz et al., 2004). Previous nPD and ICM studies examining patients with CF with a spectrum of CFTR variants with residual function demonstrated CFTR mediated ion transport in the range of about 10%–50% of normal and showed that inherent residual function greater than about 10% of normal may be associated with long-term exocrine pancreatic sufficiency (Hirtz et al., 2004; Wilschanski et al., 2006; Stanke et al., 2008; Derichs et al., 2010). This may explain why LUM/IVA therapy even led to an improvement in faecal elastase in younger children in Phase three trials (McNamara et al., 2019). Taken together, these data suggest that the degree of functional rescue achieved with LUM/IVA may improve long-term clinical outcomes in patients with CF who are F/F. The findings on the higher efficacy of LUM/IVA therapy in childhood can therefore also be seen as an argument for starting treatment in early childhood, even if short-term clinical improvements in these patients are not always immediately observable (Milla et al., 2017; Ranganathan et al., 2017; McNamara et al., 2019; Stahl et al., 2021). However, whether pharmacological rescue of CFTR function to 30% of normal CFTR activity has an impact on the clinical course and life expectancy of patients with CF remains to be shown in longer-term prospective observational or registry studies.

Linear regression analysis revealed that the increase in cAMP response in ICM under LUM/IVA correlates well with the increasing effect of the CFTR inhibitor 172, thus providing consistent electrophysiological findings (Figure 3 A). Our analyses of the relationship between SCC and CFTR-mediated Cl-secretion determined by ICM showed no correlation but a high concordance between the two CFTR biomarkers (Figure 3B). This was also the case in our study of LUM/IVA therapy in CF patients who are F/F over 12 years (Graeber et al., 2018). We suspect that several reasons may have contributed to this result. First, the way CFTR function is represented by the two CFTR biomarkers is different. While sweat chloride is an indirect measure of salt uptake in sweat ducts, ICM is a more direct measure of CFTR-mediated chloride flux. Furthermore, the bioavailability of LUM/IVA may vary in different tissues. Nevertheless, the analyses of the relationship between SCC, nPD and ICM in the TEZ/IVA and ELX/TEZ/IVA study mentioned above showed correlations between the three CFTR biomarkers in all treatment groups (Graeber et al., 2022a). However, this may have been due to the more pronounced improvements in CFTR activity in the ELX/TEZ/IVA-treated patients. Regardless, the results of the different studies suggest that the CFTR biomarkers are not directly interchangeable, but provide complementary information about CFTR function when tested in clinical trials (Graeber et al., 2015; Graeber et al., 2018; Graeber et al., 2022a).

The cohort studied was relatively small to evaluate short-term clinical changes with initiated LUM/IVA therapy. Nevertheless, we saw improvements in LCI and weight gain of patients under real-world conditions to levels comparable to the results of the phase three trials for LUM/IVA approval (Ratjen et al., 2017; McNamara et al., 2019). Specifically, the absolute change in LCI in our study was −0.7 (Table 1, Supplementary Figure S1A), compared with −1.0 in the cohort of children with CF aged 6–11 years (Ratjen et al., 2017) and −0.58 in the cohort of children with CF aged 2–5 years (McNamara et al., 2019). Consistent with this, a substantial concordance, but no correlation, was observed in the study sub-cohort between improvement in CFTR function, as measured by the two CFTR biomarkers SCC and ICM, and LCI as a significantly improved clinical parameter (Supplementary Figures S1B, C). These data suggest that clinical outcome is influenced by numerous factors independent of CFTR function, such as fixed airflow limitation due to irreversible structural lung damage, which may influence and mask improvement in clinical parameters, especially considering the relatively moderate degree of functional correction of CFTR function achieved with LUM/IVA. Furthermore, no improvement was seen in other lung function parameters, such as FEV1%predicted. BMI, which was also included in the phase-3 trials in children, also showed no significant improvement in our study cohort of children with CF aged 2–11 years on LUM/IVA therapy. However, the increase in body weight over the age-adjusted weight percentiles was significant in our study cohort (+6.3 percentile; *p*-value 0.02; Table 1). Thus, this study, as well as previous LUM/IVA clinical trials, shows that the heterogeneity in clinical endpoints is very high (Boyle et al., 2014; Wainwright et al., 2015; Konstan et al., 2017; Ratjen et al., 2017; Graeber et al., 2018). However, as the CFTR biomarkers SCC, nPD and ICM also showed much lower heterogeneity in our previous studies on IVA monotherapy and combination therapies with LUM/IVA, TEZ/IVA and ELX/TEZ/IVA in CF patients older than 12 years, we conclude that CFTR biomarkers are more sensitive than the established clinical endpoints FEV1%predicted and BMI to detect partial functional rescue of mutant CFTR by CFTR modulator therapy (Graeber et al., 2015; Graeber et al., 2018; Graeber et al., 2022a).

This study has several limitations. First, similar to previous observational studies on the effects of CFTR modulators, which were also conducted after the marketing authorization of the respective drug (Rowe et al., 2014; Graeber et al., 2015; Boutin et al., 2018; Ronan et al., 2018; Graeber et al., 2022b), it was not possible to include a placebo group in our study. However, the results of our CFTR biomarker measurements at baseline are consistent with previous ICM studies in independent cohorts of patients with CF who are F/F (Bronsveld et al., 2000; Bronsveld et al., 2001; Mall et al., 2004; van Barneveld et al., 2010; Graeber et al., 2018). In addition, all functional measurements were performed in a strictly paired manner to reduce background variability that may arise from unpaired comparison of CFTR biomarker measurements in different patients.

Second, in contrast to our previous study, we did not perform nPD examinations in this study, because in our experience this examination can only be performed reliably in children aged 8–9 years and older. We are aware that there are groups that perform these examinations in younger patients under sedation [e.g., (Sermet-Gaudelus et al., 2010)], but such sedation makes the procedure even more invasive, considering that the drug is already on the market. Therefore, based on the experience of good agreement between nPD and ICM measurements in the previous study, we had decided not to perform nPD in this age group.

Third, the conclusions that can be drawn from our data about the relationship between the extent of functional rescue of CFTR protein and improvement in clinical outcome are limited by 1) the small number of patients available for this study and 2) the limited duration of clinical follow-up. Therefore, evidence from longitudinal studies in larger cohorts, including patients with CF who are F/F, as well as patients with CFTR gating and residual function variants treated with different CFTR modulator therapies, is needed to further understand the relationship between the extent of functional rescue of CFTR protein and clinical response, including the long-term impact on disease progression and survival.

In summary, this study demonstrates for the first time that LUM/IVA treatment consistently induces partial rescue of CFTR function in F508del homozygous patients with CF aged 2–11 years, although clinical outcome parameters showed only partial improvement after a relatively short course of treatment. With LUM/IVA therapy, ICM in these young patients demonstrated functional rescue of F508del-CFTR in the intestinal epithelium at a level of approximately 30% of normal CFTR function, which is comparable to the CFTR activity previously observed in pancreas sufficient patients with CF who have a spectrum of CFTR variants with residual function (Hirtz et al., 2004; Stanke et al., 2008). With the approval of more effective CFTR modulators than LUM/IVA for this age group, further studies are needed to evaluate the rescue of CFTR activity. The CFTR biomarkers SCC and ICM used in this study appear to be well suited for future research to assess the relationship between therapeutic rescue in CFTR function and long-term clinical outcomes in children with CF.

## Data availability statement

The raw data supporting the conclusion of this article will be made available by the authors, without undue reservation.
