# Supplementary figures and images for: Effects of lumacaftor—ivacaftor therapy on cystic fibrosis transmembrane conductance regulator function in F508del homozygous patients with cystic fibrosis aged 2–11 years
Source: Front Pharmacol. 2023 May 30;14:1188051. doi: 10.3389/fphar.2023.1188051 (PMC10266342; doi:10.3389/fphar.2023.1188051)

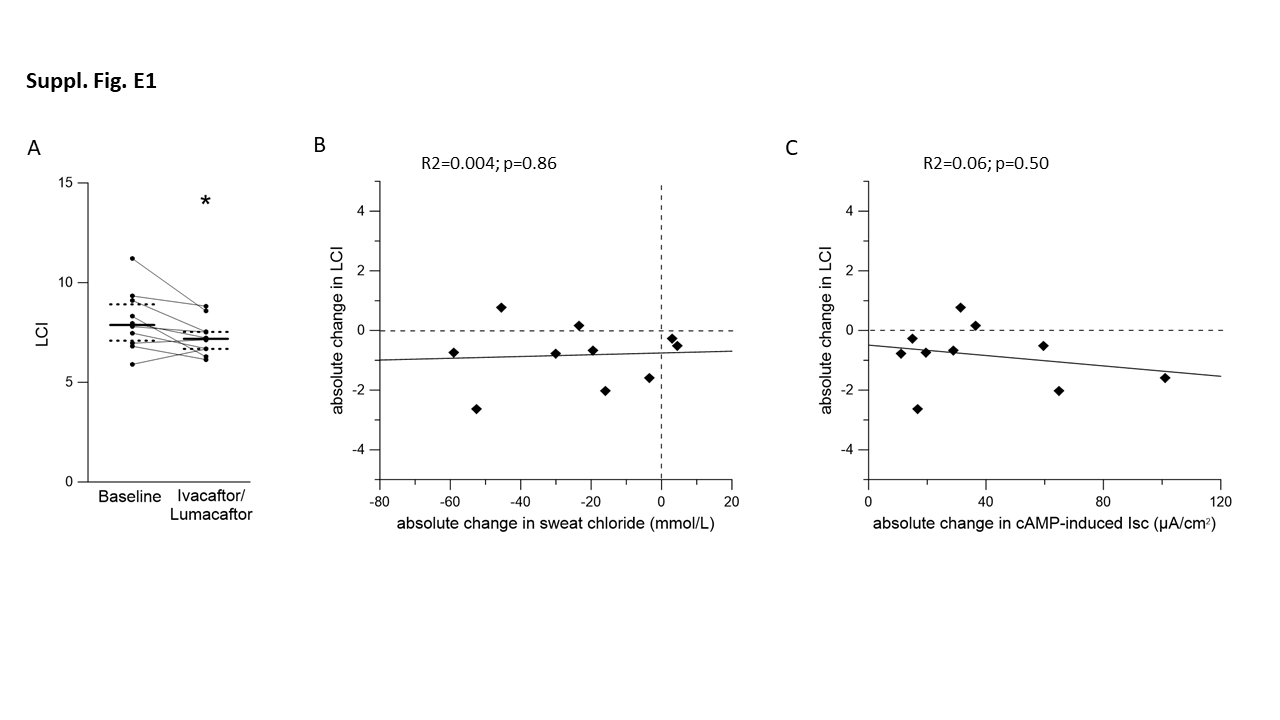

Supplement: Supplementary file 1 [file Image1.TIF]
