# Supplementary material for: Effects of lumacaftor—ivacaftor therapy on cystic fibrosis transmembrane conductance regulator function in F508del homozygous patients with cystic fibrosis aged 2–11 years
Source: Front Pharmacol. 2023 May 30;14:1188051. doi: 10.3389/fphar.2023.1188051 (PMC10266342; doi:10.3389/fphar.2023.1188051)
Supplement: Supplementary file 2 [file DataSheet1.docx]

**Online Supplement**

**Methods**

***Intestinal current measurements***

4 samples were obtained from each patient at each measurement. The samples were immediately stored in tissue medium (medium 199 containing Hank’s salts, L-glutamine and 25 mM HEPES complemented with 5 mM glycine and 0.5 mM sodium-DL-β-hydroxybutyrate or RPMI-1640 medium with L-glutamine and sodium bicarbonate) and mounted in custom made perfused micro-Ussing chambers. Quality control on biopsies allowed for usage of a median of 3.5 (range 1 to 4) tissue samples per measurement. The luminal and basolateral compartments were perfused continuously with a buffer solution of the following composition: 145 mM NaCl, 0.4 mM KH2PO4, 1.6 mM K2HPO4, 5 mM D-glucose, 1 mM MgCl2, 1.3 mM Ca-gluconate, pH 7.4, at 37°C. Experiments were performed under open circuit conditions. Transepithelial voltage (Vte) was recorded and transepithelial resistance (Rte) was determined by applying intermittent (1 s) current pulses (∆I = 0.5 µA). The equivalent short circuit current (I_eq_) was determined from continuous Vte and Rte recordings according to Ohm’s law (I_eq_ = Vte / Rte) after appropriate correction for fluid resistance (39, 40, 41). Amiloride was administered luminally (10 µM) to block electrogenic Na+ absorption, indomethacin was administered basolaterally to inhibit prostaglandin E2 synthesis as well as cAMP production. Lumen-positive (Cl-secretory) I_eq_ responses induced by cAMP-dependent stimulation with 3-isobutyl-1-methylxanthine (IBMX, 100 μM) and forskolin (1 μM) basolaterally were measured. Cholinergic co-activation with carbachol (CCH; 100 μM, basolateral) increases the intracellular Ca2+ and stimulates the basolateral Ca2+-dependent K+ channels, thus creating an electrical driving force for further luminal Cl-secretion via CFTR in non-CF individuals. In CF however, there is an inverse lumen-negative I_eq_ response reflecting luminal K+ secretion, while the lumen-positive Cl-secretory response is absent or reduced. Additionally, effects of the CFTR inhibitor-172 (20 µM, basolateral) were evaluated.

***Lung Function Test***

The Forced Expiratory Volume in one second as a percent of the predicted value (ppFEV1) was determined with spirometry and calculated according to reference values from the European Respiratory Society (11). In addition, Lung Clearance Index (LCI) representing a parameter to measure ventilation inhomogeneities in the small airways, was determined using the multiple breath washout (MBW) technique with N2 as tracer gas (15, 42). The measurements were performed with the Exhalyzer D system (Eco Medics, Duernten, Switzerland) and 100% oxygen was used to wash out resident nitrogen from the lungs with a mouthpiece as interface (43). All measurements were evaluated centrally using spiroware 3.3.1 (Eco Medics, Duernten, Switzerland) (16). The normal values for the LCI we used are reported to range from 6.16 to 7.91 for school-aged children 6 to 18 years of age (44).

***Multiple breath washout (MBW)***

MBW testing was performed with the Exhalyzer D system/spiroware 3 (Eco Medics). Pure oxygen was used to wash out resident nitrogen from the lungs as previously described (43). The LCI_2.5_ was determined from washout curves with no evidence of leak.

**Suppl. Figure E1. Significant decrease of the Lung Clearance Index (LCI); linear regression analysis reveals no quantitative correlation between CFTR biomarkers and LCI**

**A)** Paired values before and after the start of LUM/IVA treatment for LCI (**A**; median of differences -0.7, p-value 0.037). Statistical evaluation by means of Wilcoxon matched-pairs signed rank test. **B, C)** Depicted is the relationship between change in LCI as clinical parameters and sweat chloride levels (**B**) as well as cAMP response in ICM (**C**) as CFTR biomarkers. Neither for sweat chloride (**A**: R2=0.004; p=0.86), nor for ICM data (**B**: R2=0.06; p=0.50) a significant positive correlation could be established.
